# Determinants of basic public health services provision by village doctors in China: using non-communicable diseases management as an example

**DOI:** 10.1186/s12913-016-1276-y

**Published:** 2016-02-04

**Authors:** Tongtong Li, Trudy Lei, Zheng Xie, Tuohong Zhang

**Affiliations:** 1 School of Public Health of Peking University, 38 Xueyuan Road, Haidian District, Beijing, P. R. China; 2 School of Public Health of Columbia University, New York, USA

**Keywords:** Village doctors, Equity, Primary health care

## Abstract

**Background:**

To ensure equity and accessibility of public health care in rural areas, the Chinese central government has launched a series of policies to motivate village doctors to provide basic public health services. Using chronic disease management and prevention as an example, this study aims to identify factors associated with village doctors’ basic public health services provision and to formulate targeted interventions in rural China.

**Methods:**

Data was obtained from a survey of village doctors in three provinces in China in 2014. Using a multistage sampling process, data was collected through the self-administered questionnaire. The data was then analyzed using multilevel logistic regression models.

**Results:**

The high-level basic public health services for chronic diseases (BPHS) provision rate was 85.2 % among the 1149 village doctors whom were included in the analysis. Among individual level variables, more education, more training opportunities, receiving more public health care subsidy (OR = 3.856, 95 % CI: 1.937–7.678, and OR = 4.027, 95 % CI: 1.722–9.420), being under integrated management (OR = 1.978, 95 % CI: 1.132–3.458), and being a New Cooperative Medical Scheme insurance program-contracted provider (OR = 2.099, 95 % CI: 1.187–3.712) were associated with the higher BPHS provision by village doctors. Among county level factors, Foreign Direct Investment Index showed a significant negative correlation with BPHS provision, while the government funding for BPHS showed no correlation (P > 0.100).

**Conclusion:**

Increasing public health care subsidies received by individual village doctors, availability and attendance of training opportunities, and integrated management and NCMS contracting of village clinics are important factors in increasing BPHS provision in rural areas.

**Electronic supplementary material:**

The online version of this article (doi:10.1186/s12913-016-1276-y) contains supplementary material, which is available to authorized users.

## Background

Village doctors are on the front lines of health care provision [1] and, at one point in time, greatly improved the equity and accessibility of public health care in rural China. A three-tiered rural health system was established in the 1960s [2], where village clinics serve the bottom-tier. The “barefoot doctor” training program was implemented to create a primary workforce for public health care [3] and provide basic medical services [4] to rural populations. These barefoot doctors were local farmers who were recruited, trained, and supported through the Cooperative Medical Scheme (CMS) [5]. CMS was a highly collective medical organization, operated privately by the barefoot doctors [6] and subsidized by public funds of local people’s communes. Under such situation, barefoot doctors worked as half-peasant-half-doctor with limited medical knowledge, but they alleviated the shortage of medicine successfully. By the 1970s, they improved the quality and accessibility of health care in rural China, which was deemed by the World Health Organization’s Declaration of Alma-Ata to be an international model for providing primary health care [7].

However, along with economic reform during the early 1980s, the barefoot doctor system ceased and local governments took over the provision of rural public health care services. Due to the collapse of the CMS, the central government dramatically reduced health care funding in rural areas [8]. The barefoot doctors became unemployed and those who passed a qualifying examination reemerged as “village doctors” as designated by the Ministry of Health [9]. No longer were there effective incentives encouraging village doctors to provide unprofitable public health care services [10, 11]. Instead, the economic reform forced village doctors to seek income in other ways, specifically, shifting their focus to fee-for-service medical activities [12] and profits from the 15 % markup on prescribed drugs dispensed to patients [13]. Though village doctors still served as the bottom-tier of the rural health system, the government did not provide any models or guidelines to help support them through the changes to health service provision [14]. Thus, the changes brought about by privatization left local residents holding lower respect of village doctors. Since supporting basic public health services became the responsibility of local governments and due to the urban versus rural economic disparity, health equity declined greatly [15–18]. Residents in rural areas often struggled to meet their health care needs and suffered from heavy disease burden [19, 20]. It became a serious threat to the health of rural residents in China [21–24].

Providing good quality and accessible public health care for rural population is an important issue for Chinese healthcare reform [25]. The Chinese government launched the Health Sector Reform in 2009 with one of the goals being to provide a package of basic public health services including health records creation for every resident, health education, immunization, chronic disease (hypertension and diabetes) management, severe mental diseases patient management, maternal and child health care services, elderly health care, and so forth [26]. However, there are still significant provision variations among different basic public health items. As an example, previous studies have showed that, for immunization as well as maternal and child health care services [27,28] were traditionally well provided by village doctors, while the provision of chronic disease management and prevention was extremely low [29]. These newly added items of basic public health services for chronic diseases (abbreviated here as BPHS) increased the workload of village doctors naturally. In practice, however, the quantity and quality of BPHS provision vary among different village doctors [26, 30].

To achieve the goal of providing basic public health services, the Health Sector Reform also formulated a series of strategies to “build a strong rural health service network,” such as providing more training for, strengthening management to, and improving subsidies for village doctors [31]. Providing routine training programs is the first requirement for increasing provision and improving consistency of BPHS. Content of such trainings include health care policy, standards, and BPHS quality management. As most village doctors begin to work immediately after obtaining their secondary school degree [32], on-the-job training is essential to ensure the quality of their services [33], especially regarding BPHS [30]. Fortunately, since the Health Sector Reform in 2009, the central government has required township health centers (THCs) to devise structured and tailored training programs to update village doctors’ knowledge and techniques on public health services.

There are also other new political strategies incentivizing and supervising BPHS provision by village doctors. In 2003, the Chinese government initiated the New Cooperative Medical Scheme (NCMS), a government-run voluntary insurance program for rural residents [34], which has covered almost all of the targeted population in China since 2010 [35]. However, not all village clinics are NCMS-contracted providers, thereby possibly influencing patient choice of health care provider due to availability of reimbursements, which would affect BPHS provision by village doctors [5]. Also, in 2009, integrated management was established, that is, the THCs began take on responsibility for the management of the village clinics, including medicine, personnel, finance, facilities, routine work, etc. Additionally, all work related to the public health services of village doctors was under the supervision and management of THCs, which significantly affected village doctors’ income structure [5] and pushed a shift in focus of their daily work from medical services toward public health.

Lastly, subsidies given to village doctors’ subsidies on BPHS is also one major strategy carried out since the Health Sector Reform. The central government created a specific fund, the “basic public health service fund”, allocated to the THCs for distribution to village doctors to motivate them to provide basic public health services. The central government dictated an increase in amount of the funds from ¥25 (US $4.033) per person in the service population in 2012 to ¥40 (US $6.453) in 2015. However, the actual subsidy received per person differs by county since local government may also add to the fund provided by the central governmental according to the local fiscal status. While the subsidy in some counties remains at national standard with no local government supplementation, in some eastern counties with sufficient supply from local financing, it reaches ¥100 (US $16.132). Furthermore, there are also some other factors affecting village doctors’ behavior collectively. For example, village doctors living in different geographic counties or counties with different levels of economic openness may hold different views on the provision of BPHS.

Village doctors’ personal BPHS provision is deeply affected by the county-level factors, as well as by individual characteristics. However, limited studies concerning both level factors are available. Hence, this study aimed to investigate the factors associated with village doctors’ BPHS provision at both individual and county levels and to provide possible policy recommendations to improve public health care equity and accessibility in rural area.

## Methods

### Data

**Fig. 1:**
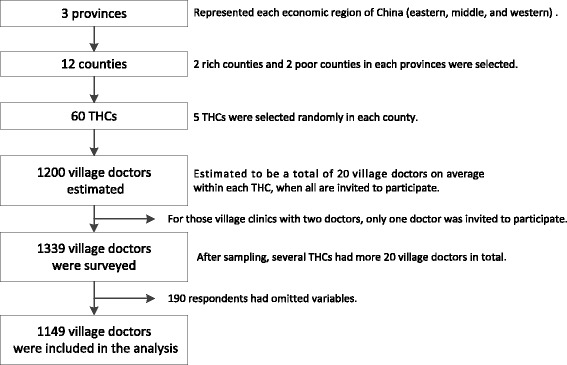
Flow chart for the sampling

This study used a multi-stage sampling design. First, three provinces were selected to represent each economic region of China (eastern, middle, and western) as previously defined by the government. Within each province, counties were designated as rich or poor based on available socioeconomic status data, then two rich counties and two relatively poor counties were chosen randomly within each designation. Health care managers helped to call all local village doctors to the THCs on the day of visit. Each THC governed approximately 20 village clinics, and each clinic generally had one village doctor (for those have two doctors, only one doctor was invited to participant), totaling 20 village doctors on average in each THC. The research team visited the selected THCs, and all village doctors present were invited to participate in the survey. To ensure confidentiality, no respondent identifiers were recorded. All respondents finished their questionnaires on their own, but research staffs were available and ready to address any questions raised by respondents. All eligible village doctors agreed to participate. The final sample consisted of 1149 village doctors in 12 county-level units with between 100 and 140 village doctors representing each county (Fig. 1). Ethics approval for this study was obtained from Peking University Health Science Center in China (protocol number IRB00001052- 14017).

### Measures

#### Dependent variables

Provision of BPHS by village doctors was assessed using a questionnaire that included three items: “Do you manage hypertension diseases for local residents?”, “Do you manage diabetes for local residents?”, and “Do you create health records for all citizens?”, all with the response categories “yes” and “no”. Responses were given a value of 1 for “yes” and 0 for “no”, and the sum score thus ranged from 0 to 3. As all three are essential BPHS services [36], village doctors who scored 0, 1, or 2 were considered to be low-level BPHS providers (coded 0) and village doctors with scores of 3 were considered to be high-level providers (coded 1).

#### Individual-level variables

Participation in training programs was measured by: “How many times did you participate in village doctors training programs during the last 3 years?” Monthly public health care subsidy received was measured by: “How much do you earn monthly from the government for public health services?” Three categories were created from the responses: none (0), 1–300 Chinese Yuan (CNY) (1), and more than 300 CNY (2).

Integrated management was measured by three questions: “Do THCs manage the finances of your village clinic?”, “Do THCs manage the personnel salary of your village clinic?”, and “Do THCs manage medical drugs of your village clinic?” Responses for each were recorded on a three-point scale: “total”, “partial”, and “none”. Responses were given a value of 2 for “total”, 1 for “partial,” and 0 for ‘none’, and the sum score ranged from 0 to 6. Village clinics, which scored 0, 1, 2, or 3, were considered as low level of integrated management (coded 0), while village clinics with scores of 4, 5, or 6 were considered high level (coded 1).

NCMS-contracted was measured by one question: “Is your village clinic a NCMS-contracted medical institution?” with response categories ‘yes’ and ‘no’. Responses were given a value of 1 for ‘yes’ and 0 for ‘no’.

Control variables included age (20–39, 40–59, and 60 or over), gender, and education level (completion of junior high school or less, completion of secondary school, higher than secondary school). Participants’ average monthly income ranged from 0 to 4000 CNY and was separated into three categories of equal proportion: low, average, and high (Additional file 1).

#### County-level variables

Three county-level variables were included in the analysis: (i) geological characteristics, (ii) Foreign Direct Investment Index (FDI Index), the ratio between foreign direct investment and gross domestic product (GDP) in 2013, revealing the openness of the local economy [37], and (iii) funding of BPHS, from central and local government budgets, expressed as funding for each local resident (Additional file 2).

### Analyses

All analyses were conducted using STATA version 12.0. Descriptive statistics were used to describe the characteristics of the study population. To determine the differences in individual variables according to BPHS provision, Chi-squared tests and t-tests were performed. To model the effects of compositional (individual level) and contextual (county level) variables on BPHS provision by village doctors, the data was fitted using a logistic regression with village doctors’ reported provision of BPHS as the outcome. Odds ratios (ORs) and their 95 % confidence intervals (CIs) for BPHS provision were analyzed using multilevel logistic regression models, adjusting for both individual and county level variables as fixed effects and allowing for heterogeneity between counties. A series of five models was performed with Model 1 as a null model containing no explanatory variables. Intra-class correlation coefficient (ICC) was computed to examine the necessity of fitting multilevel models. Model 2 included all the control confounders at the individual level. Model 3 and Model 4 added individual and county level variables, respectively, into Model 2. Model 5 added both individual and county level variables into Model 2. Comparing Models 3 through 5, the impacts of compositional and contextual variables on provision of BPHS and their changes after controlling for each other were assessed.

## Results

### Descriptive statistics of the sample

At the individual level, a considerable number of respondents were male (75.2 %), were between 40 and 59 years old (53.1 %), held a secondary school degree (68.1 %), and earned a monthly subsidy between 1 and 300 CNY for public health service (59.0 %). On average, each village doctor had attended 12.7 (±8.791) trainings in the last 3 years. In regards to central policies, 671 (52.4 %) village doctors worked in a clinic with high-level integrated management and 787 (68.5 %) belonged to NCMS-contracted provider clinics. At the community level, more than half of the counties were in the mountain area (62.5 %) and 64.1 % got 30 CNY of BPHS funding per resident (Table 1).

**Table 1:** Descriptive statistics

Variable	Sample	Percentage (%)
Individual level (level-1, *n* = 1149)		
Gender		
Male	864	75.2
Female	285	24.8
Age (years)		
20–39	344	29.9
40–59	610	53.1
60 +	195	17.0
Education		
≤ Junior high school	149	13.0
Secondary school	783	68.1
> Secondary school	217	18.9
Average monthly income (CNY) ^b^		
Low	392	34.1
Ordinary	377	32.8
High	380	33.1
Frequency of training in last 3 years	1149	12.7 (8.791)^a^
Monthly public health care subsidy (CNY) ^b^		
None	159	13.8
1–300	678	59.0
More than 300	312	27.2
Integrated Management		
Low	561	47.6
High	671	52.4
NCMS-contracted		
No	362	31.5
Yes	787	68.5
County level (level-2, *n* = 12)		
Geographical factor ^c^		
Mountain area	8	62.5
Plain	4	37.5
FDI Index ^d^		
Low	5	38.6
Average	3	28.9
High	4	32.6
Funding for BPHS per person (CNY) ^b, c^		
Less than 30	2	14.5
30	8	64.1
More than 30	2	21.4

^a^ Training opportunity last 3 years is a continuous variable, presented by mean (S.D.) instead of percentage

^b^ USD $1 = CNY 6.199 (June 1, 2015)

^c^ Aggregate variables

^d^ Integral variables

### Differences in individual-level characteristics of BPHS provision

Among 1149 participants, 979 (85.2 %) village doctors provided high-level BPHS (Table 2). The high BPHS group had more men and more members between 40 and 59 years old in comparison to those in the low BPHS group. Members of the high BPHS group had received more education and training than those in the low BPHS group. Those who earned less in total or earned less public health subsidy were more likely to belong to the low BPHS group. Village doctors who worked in NCMS-contracted village clinics and high-level integrated management more likely belonged to the high BPHS group (*P* < 0.001).

**Table 2:** The differences of individual level characteristics by BPHS provision in China

Variables	Low-level BPHS (*n* = 170) Percentage (%)	High-level BPHS (*n* = 979) Percentage (%)	*P* value ^b^
Gender			0.002
Male	65.9	76.8	
Female	34.1	23.2	
Age (years)			0.030
20–39	30.6	29.8	
40–59	45.9	54.4	
60 +	23.5	15.8	
Education			<0.001
≤ Junior high school	24.1	11.0	
Secondary school	65.3	68.6	
> Secondary school	10.6	20.3	
Average monthly income (CNY) ^c^			<0.001
Low	49.5	31.5	
Ordinary	22.9	34.5	
High	27.6	34.0	
Frequency of training in last 3 years ^a^	11.5	12.9	0.049
Monthly public health care subsidy (CNY) ^c^			<0.001
None	17.6	13.2	
1–300	74.2	56.4	
More than 300	8.2	30.4	
Integrated Management			<0.001
Low	82.4	43.0	
High	17.6	57.0	
NCMS-contracted			<0.001
No	70.0	24.8	
Yes	30.0	75.2	

^a^ Training opportunity last 3 years is a continuous variable, presented by mean instead of percentage

^b^ *P* value by chi-square test in categorical variables and *t*-test in continuous variables

^c^ USD $1 = CNY 6.199 (June 1, 2015)

**Fig. 2:**
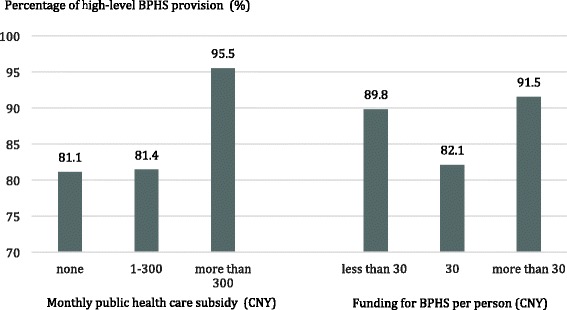
Village doctors’ BPHS provision level under different subsidies in China

Figure 2 presents some findings in a histogram (based on percentage of village doctors providing high-level BPHS). The percentage of high-level BPHS provision in relation to public health care monthly subsidies showed a positive trend with increased subsidy. Yet there is no significant trend regarding the percentage of high-level BPHS provision versus government funding for BPHS per person.

### Multilevel logistic regression estimates of BPHS provision

Table 3 shows the results of multilevel logistic regression analysis models testing the individual and county level factors associated with BPHS provision. Without including any explanatory variables, 38.4 % of the variance in village doctors’ BPHS provision came from the county level and there was significant difference among counties (Model 1). After adding confounders, the county level variance decreased, but still remained significant (Model 2).

**Table 3:** Multilevel logistic regression estimates and variance components of provision of BPHS of village doctors, N = 1149 individuals nested within *N* = 12 counties

Variables	Model 1		Model 2		Model 3		Model 4		Model 5	
	OR	95 % C.I.	OR	95 % C.I.	OR	95 % C.I.	OR	95 % C.I.	OR	95 % C.I.
Individual level										
Gender										
Male (ref)										
Female			0.828	(0.525,1.304)	0.842	(0.521,1.360)	0.840	(0.533,1.322)	0.860	(0.533,1.387)
Age (years)										
20–39 (ref)										
40–59			1.433	(0.908,2.262)	1.467	(0.904,2.381)	1.445	(0.916,2.281)	1.474	(0.908,2.390)
60 +			1.495	(0.744,3.003)	1.697	(0.802,3.594)	1.494	(0.745,2.996)	1.685	(0.799,3.555)
Education										
≤ Junior high school (ref)										
Secondary school			1.694*	(0.905,3.171)	1.974**	(1.015,3.838)	1.681	(0.899,3.143)	1.926*	(0.993,3.737)
> Secondary school			2.628**	(1.160,5.955)	3.459***	(1.438,8.317)	2.656**	(1.173,6.012)	3.443***	(1.434,8.266)
Average monthly income										
Low (ref)										
Ordinary			1.853**	(1.104,3.111)	1.379	(0.796,2.389)	1.841**	(1.098,3.087)	1.351	(0.781,2.336)
High			1.110	(0.680,1.812)	0.992	(0.584,1.687)	1.114	(0.684,1.817)	0.978	(0.577,1.660)
Frequency of training in last 3 years					1.034**	(1.002,1.066)			1.034**	(1.003,1.066)
Monthly public health care subsidy (CNY)										
None (ref)										
1-300					3.546***	(1.795,7.004)			3.856***	(1.937,7.678)
More than 300					3.886***	(1.683,8.972)			4.027***	(1.722,9.420)
Integrated Management										
Low (ref)										
High					1.911**	(1.096,3.332)			1.978**	(1.132,3.458)
NCMS-contracted										
No (ref)										
Yes					2.005**	(1.131,3.554)			2.099**	(1.187,3.712)
County level										
Geographical factor										
Mountain area (ref)										
Plain							1.995	(0.390,10.201)	2.761	(0.602,12.671)
FDI Index										
Low(ref)										
Average							1.276	(0.172,9.470)	1.498	(0.239,9.394)
High							0.159**	(0.032,0.784)	0.289*	(0.071,1.179)
Funding for BPHS per person (CNY)										
Less than 30 (ref)										
30							0.726	(0.098,5.370)	0.324	(0.051,2.045)
More than 30							0.334	(0.030,3.706)	0.233	(0.026,2.086)
Variance components										
County level variance	2.047***		1.957***		1.604***		1.118***		0.772***	
Intra-class correlation	0.384		0.373		0.328		0.254		0.190	

*P < 0.100; **P < 0.050; ***P < 0.010

In Model 3, village doctors who received more education (OR = 1.974, 95 % CI: 1.015–3.838, and OR = 3.459, 95 % CI: 1.438–8.317), more training opportunities (OR = 1.034, 95%CI: 1.002–1.066), and more public health care subsidy were more likely to provide high BPHS. More specifically, village doctors who received 1–300 CNY public health care subsidy monthly provided more BPHS than those who received no subsidy (OR = 3.564, 95 % CI: 1.795–7.004) and those who received even more than 300 CNY per month provided even more services (OR = 3.886, 95 % CI: 1.683–8.972). Additionally, integrated management (OR = 1.911, 95 % CI: 1.096–3.332) and NCMS contracting (OR = 2.005, 95 % CI: 1.131–3.554) increased the probability of high BPHS provision of village doctors. The results were quite constant even after including county level variables (Model 5).

In Model 4, increased FDI index at the county level reduced the probability of high BPHS provision. Government funding for BPHS was not associated with village doctors’ level of BPHS provision. After including individual level factors, the influence of contextual variables remained unchanged (Model 5), though the exact value of OR and CI changed slightly. Furthermore, from Models 2 to 5, the county-level variance decreased by 62 %, which implied that these variables had good explanatory power for the variance in village doctors’ BPHS provision.

## Discussion

Our findings show that village doctors with higher education levels and more training opportunities were more likely to be high BPHS providers, consistent with prior studies in China [36, 38]. This may be because those with higher education levels and more training opportunities have better knowledge of BPHS [33, 39, 40]. Before 2009, most of the training developed by local health departments for village doctors focused on disease treatment, clinical skill, and basic health care policy [41]. However, since the Health Sector Reform, the central government has shifted the focus of training to primary health care [42], especially the norms, standards and service delivery paths of BPHS, as recommended by international studies as an effective way to improve public health services in rural areas [43, 44]. However, due to local economic limitations or personal choice, these training programs were still not necessarily available for and attended by all the village doctors in China [38]. Thus, to improve BPHS provision by village doctors, education of and training opportunities for village doctors should be increased, with a focus on knowledge about public health services.

The results show that monthly BPHS subsidies at the individual level exhibit the most consistently positive correlation with the BPHS outcome. This means that financial incentives still play an essential role in scaling up BPHS provision by village doctors. Although China has grown to be the second largest economy in the world [45], with the increase in income per capita, village doctors still earn a relatively low income. Their income is not only lower than those working in THCs [46], but also lower in respect to their own income of previous years [30, 47, 48], due to the switch to the fee-for-service payment model after the economic reform in the 1980s. Therefore, without any financial support from the government, they have little incentive to provide public health services [49]. Thus, the special subsidy for BPHS, about ¥200–400 (US $32.263–64.527) per month, is a crucial component of their total income and a strong motivator for providing BPHS to local residents.

Compared to BPHS subsidies at the individual level, the government funding for each resident within the service population on the county level shows no resulting difference in BPHS provision by village doctors. This may be due to inconsistency in the availability and amount of government funding received by village doctors. First of all, this government funding of BPHS for local residents is based on both central and local government finance and varied in different areas depending upon local fiscal capacity [42]. For example, the government funding for BPHS in Beijing is triple the national standard [50], while in other provinces it is near or lower than national standard. This is one major obstacle to providing high-level public health services in many rural areas [51]. Secondly, although THCs are required to allocate no less than 40 % of government funding of BPHS to village doctors, the specific proportion actually received is uncertain [48]. A previous study in China showed only a small portion is used to compensate village doctors [26], which would fail to motivate village doctors to provide public health services. Thus, more funding should be allocated to village doctors directly in order to improve BPHS provision in rural China long-term.

Village clinics under high-level integrated management and NCMS contracting were also more likely to provide high provision of BPHS by village doctors after controlling for other factors. This may be due to the supervision and support of integrated management as well as the outpatient reimbursement of NCMS [52]. Integrated management is a newly implemented policy in the rural health care service scheme, aiming to manage village doctors as ordinary primary health care providers under THCs. Under this policy, village doctors are not only supervised by the public health service standards of THCs [53], but also supported by advantageous knowledge and resources through contracts with THCs [54]. Since the integrated management policy’s creation in 2009, it has expanded considerably and may cover all village clinics in the future. The NCMS insurance program includes considerable compensation for outpatient care of rural residents and is heavily subsidized by central, provincial, and county governments [55]. NCMS covers care at THCs and county-level hospitals, which are public, government-owned institutions in China. The village clinics, however, are not all covered by this policy. Our data showed that only 68.5 % village clinics among the total surveyed were NCMS-contracted medical institutions. Consequently, patients who do not live near any NCMS-contracted village clinics would be motivated by NCMS outpatient reimbursement rates to go to THCs and county-level hospitals for minor illnesses rather than to their village clinics [56]. Thus, village doctors who work without contracts with NCMS would lose their opportunities to provide BPHS and face more challenges to ensure the public health care of local residents. Fortunately, both integrated management and the NCMS are major policies of the Health Sector Reform in China with a trend towards expanded coverage of more village clinics in the future. Together, these changes show positive steps in policy advancement toward the improvement of BPHS provision by village doctors.

Like other rapidly developing countries, China is experiencing an increase in chronic health issues along with the resulting social and economic burdens [57, 58]. However, before the Health Sector Reform in 2009, chronic disease prevention and management received relatively less attention compared with other basic public health services. Traditional public health care services such as immunization and maternal- and child-care have experienced numerous intervention projects and have been well-provided nationwide for a long period [59], while chronic disease prevention and management is only a newly added item with a low starting point towards achieving accessibility and equity of provision, especially in rural areas. Fortunately, the central government and medicine relative organizations in China have already begun to increase awareness of this issue and strengthen support gradually. However, our results showed that the coherence of current chronic disease prevention and management policies are not ideal, with space for improvement as discussed above. Thus, other political, financial, and material support is necessary to improve the remuneration and mobilization of village doctors, and realize the goal of public health service equalization eventually.

There are several limitations in this study. Firstly, although the selected provinces are generally representative of the typical economic and health development characteristics in China, the study area is limited to three provinces, which may compromise the generalizability of the findings. A larger geographical area would have more external validity. Secondly, the cross-sectional nature of the study dictates that only correlation, rather than causation, can be studied. In the future, it would be useful to perform a more comprehensive study to further research financial incentives of village doctors’ BPHS provision and how health care policies influence them.

## Conclusion

There is considerable room for improvement regarding the factors associated with village doctors’ BPHS provision. Specifically, three key areas have been elucidated in order to increase BPHS provision in rural areas: (i) increasing public health care subsidies for village doctors and ensuring transparency in the allocation of government funding to village clinics, (ii) mobilizing resources and village doctors to provide and attend ample training programs, and (iii) expanding NCMS contracting with village clinics and partnering with THCs for integrated management. Expansion and enforcement of current policies by the Chinese government to address these factors is essential toward helping to improve accessibility and quality of basic public health care in rural areas, reduce urban–rural disparities, and increase health equity nationwide. In addition, as urbanization grows globally, these findings about the Chinese health care reform experience may also be relevant for other developing countries in recognizing possible rural area health provision shortfalls and designing effective strategies to ensure public health care.

## Additional files

Additional file 1: **Questionnaire of village doctors in China.** (PDF 41 kb)

Additional file 2: **County level questionnaire of village doctors in China**. (PDF 20 kb)

## Abbreviations

BPHS: basic public health services for chronic diseases
CMS: cooperative medical scheme
CNY: Chinese Yuan
FDI Index: foreign direct investment index
GDP: gross domestic product
NCMS: new cooperative medical scheme
THCs: township health centers
