# Supplementary material for: Determinants of basic public health services provision by village doctors in China: using non-communicable diseases management as an example
Source: BMC Health Serv Res. 2016 Feb 4;16:42. doi: 10.1186/s12913-016-1276-y (PMC4743421; doi:10.1186/s12913-016-1276-y)
Supplement: Supplementary file 1 — Questionnaire of village doctors in China. (PDF 41 kb) [file 12913_2016_1276_MOESM1_ESM.pdf]

Questionnaire of village doctors in China

_____Province _____County _______Town No:_______

In order to understand the situation of village doctors in China, and the impacts of current policies on village doctors, would you spare a few minutes to answer the following questions in this questionnaire carefully and authentically? We would like to inform you that your answers will be kept strictly confidential!

School of Public Health, Peking University

May 2014

1. Do THCs manage the finances of your village clinic?
   [1] Yes, all [2] No, none [3] Yes, partially

2. Do THCs manage the personnel salary of your village clinic?
   [1] Yes, all [2] No, none [3] Yes, partially

3. Do THCs manage the medical drugs of your village clinic?
   [1] Yes, all [2] No, none [3] Yes, partially

4. Is your village clinic a NCMS-contracted medical institution?
   [1] Yes [2] No

5. The area your village clinics covers: _________m^2

6. What medical equipment do you have in your village clinics:

	Equipment	Number
6.1	Blood pressure monitor	
6.2	Height and weight meter	
6.3	Diagnosis and treatment bed	
6.4	High pressure disinfection equipment	
6.5	Blood glucose meter	
6.6	Computer	
6.7	Electrocardiogram machine	

7. How many hours do you work as a village doctor per day on average: ___________hour(s)
   Basic health services as a percentage of your total workload: ___________%

8. The population you serve is about: _______________ people

9. Does the village clinic you belong to use a single diagnostic and treatment fee system?
   [1] Yes [2] No

10. Which is preferred for villagers to visit when then get sick generally (such as: catch a cold)?
[1] village clinics [2] private clinics [3] township health centers [4] hospitals

11. Do you manage hypertension diseases for local residents?
[1] yes [2] no

12. Do you manage diabetes for local residents??
[1] yes [2] no

13. Do you create health records for all citizens?
[1] yes [2] no

14. Besides serving as village doctor, do you have another job?
[1] yes [2] no

15. Are you satisfied with your work?
[1] very satisfied [2] satisfied [3] not satisfied [4] extremely not satisfied

16. How much do you earn monthly from the government for public health services? _______________Yuan

17. Compare your income with the average of your village:
[1] lower [2] similar [3] higher [4] not clear

18. How much income do you expect on average as a village doctor annually? ___________Yuan

19. Which kind of pension plan do you have?
[1] xinnongbao [2] none [3] others___________

20. Have you ever tried to quit your job as a village doctor?
[1] yes [2] no

21. Are you proud of being a village doctor?
[1] very proud [2] proud [3] not proud [4] extremely not proud

22. Which of the following certificate(s) do you have?
[1] village doctor practitioner certificate [2] medical practitioner certificate
[3] certificate of practicing physician assistant [4] none

23. How many times did you participate in village doctor training programs during the last 3 years: _________times

24. How much total training do you think there should be within a year: _________day(s)

Personal information:

1. Gender: [1]Male [2] Female

2. Age:_________

3. Average monthly income: _____Yuan

4. Your education level:
   [1] completion of primary school or less [2] junior high school
   [3] secondary school [4] higher than secondary school

5. Major:
   [1] clinical medicine [2] public health [3] nursing
   [4] Chinese traditional medicine [5] others [6] none

6. How long have you been a village doctor? ________year(s)

7. Have your family members ever been a village doctor?
   [1] yes [2] no [3] not clear

8. Personal health status:
   [1] good [2] have chronic disease [3] disabled [4] not clear

Thank you very much for your support and cooperation!
