# Supplementary material for: Determinants of basic public health services provision by village doctors in China: using non-communicable diseases management as an example
Source: BMC Health Serv Res. 2016 Feb 4;16:42. doi: 10.1186/s12913-016-1276-y (PMC4743421; doi:10.1186/s12913-016-1276-y)
Supplement: Supplementary file 2 — County level questionnaire of village doctors in China. (PDF 20 kb) [file 12913_2016_1276_MOESM2_ESM.pdf]

County level questionnaire of village doctors in China

_____________Province __________County

No.	Index	Value
1	Geographic situation：<br>[1] mountain [2] plain	
3	Foreign direct investment in 2013 (Yuan)	
4	Gross domestic product in 2013 (Yuan)	
5	Household population in 2013	
6	Migrant population in 2013	
7	Resident population in 2013	
9	Funding for basic public health services per capita (Yuan per month)	
